# Social differences in smoking and snuff use among Norwegian adolescents: A population based survey

**DOI:** 10.1186/1471-2458-8-322

**Published:** 2008-09-22

**Authors:** Liv Grotvedt, Hein Stigum, Ragnhild Hovengen, Sidsel Graff-Iversen

**Affiliations:** 1 Department of Health Statistics, Norwegian Institute of Public Health, Oslo, Norway; 2 Department of Chronic Diseases, Norwegian Institute of Public Health, Oslo, Norway

## Abstract

**Background:**

A change in pattern of tobacco use has been observed in the last decade in Norway. Snuff use and occasional smoking have to some degree replaced daily smoking among adolescents and young adults. Daily smoking is known to be negatively associated with social background factors, but little is known about these associations for other types of tobacco use. Our aim was to study different types of tobacco use among adolescents according to gender, educational ambitions, family background factors, and urbanization.

**Methods:**

Cross-sectional, school-based study with 15 931 participants and response-rate 87%, conducted among 15 and 16 year olds during 2000–2004.

**Results:**

More girls (33.8%) than boys (26.4%) were daily or occasional smokers, while more boys (21.4%) than girls (3.5%) were daily or occasional snuff users. Daily smoking was more common among adolescents planning vocational education, with single parents or poor family economy. Occasional smoking and snuff use (daily or occasionally) showed a similar, but less pronounced pattern regarding education and single parent families. Adolescents with parents from foreign countries were less likely to use tobacco. One exception was boys with parents from Muslim majority countries who had an increased risk of daily smoking. A typical combination user of both tobacco types was a Norwegian boy with divorced parents and ambitions to complete vocational studies or only one year of upper secondary school.

**Conclusion:**

Tobacco use in adolescents is mainly associated with low educational ambitions and less affluent self-reported family economy. Adolescents with divorced parents use more tobacco than those living with both parents. Public health initiatives to avoid or reduce tobacco use should mainly target adolescents in vocational studies and those leaving school early.

## Background

During the past ten years, the sale of oral moist snuff has increased in Norway, while the sale of tobacco for smoking has decreased. Snuff use and occasional smoking have to some degree replaced daily smoking among adolescents and young adults. The snuff marketed in Norway and Sweden (snus) is a non-fermented, moist and smokeless tobacco product [1,2]. The sale of snuff is illegal in the European Union (EU), except in Sweden where the legal use is claimed to reduce the smoking rates [3-5]. Smokeless tobacco is used on a relatively wide scale in Norway, a country which is not a member of the EU.

The ban on cigarette smoking in restaurants and bars, which was introduced in Norway in June 2004, may have influenced changes in choice of tobacco type. A Norwegian national survey among pupils in lower secondary school (13–16 years) showed the prevalence of daily smoking to be 5% in 2005, which was half the rate found in the survey five years earlier. Occasional smoking decreased from 18 to 9% in the same period. Snuff use among boys did not change, showing 4% daily and 12% occasional users in 2005. An increase in occasional snuff use from 2% to 5% from 2000 to 2005 was found among the girls [6].

The use of snuff is considered to be less harmful than cigarette smoking, but the evidence of health risks is by no means consistent [7-10]. Two recent reviews on possible health effects of snuff produced conflicting results; one concluded that there is limited epidemiological evidence about the health effects, whereas the other indicated increased risk of myocardial infarct and cancer, assessing experimental evidence from animal studies in addition to research in humans. Both reports concluded, however, that snuff use causes nicotine dependence [11,12]. Combined use of snuff and cigarettes among male adolescents has been associated with higher levels of nicotine dependence than cigarettes alone [13]. Most users of snuff combine it with smoking cigarettes [14]. The International Agency for Research on Cancer stated in 1985 that there was a carcinogenetic effect of snuff, which was confirmed in 2005 [15,16].

In Western countries, daily smoking is known to be negatively associated with socio-economic status (SES) [17-22]. The association of snuff with SES is less clear. A Swedish study pointed out an increase in snuff use among well educated urban young people [12]. A similar trend has been shown for occasional smoking [23-26]. In a Swedish city, snuff use was more common among 18 years old pupils attending vocational schools than academic schools and among boys whose parents had no more than compulsory education [27]. In Sweden, regional differences have been found for snuff use, with the highest prevalence in northern rural areas [28]. In the 1980's, prevalence of snuff use was 10% daily and 23% occasional among Norwegian army conscripts, also among athletes and highly educated people [29]. Compared with smoking, the use of snuff seems to differ less by SES and more by region [11,17].

The aim of this study is to describe the use of tobacco in 15–16 year old pupils by gender, educational ambitions, family background factors, and urbanization. In particular, this study aims to improve knowledge of socio-economic differences in snuff use and combination use of snuff and smoking. Considering that Nordic countries are in the late stages of the smoking epidemic, we expected to find marked SES differences in the prevalence of daily smoking in our study [18,20]. Little is known, however, about the extent of SES differences in adolescents' occasional smoking and snuff use, which may both represent tobacco use epidemics that differ from daily smoking. Based on existing literature in older age groups, we would expect less SES difference for occasional smoking and snuff use than for daily smoking, or even a positive association between SES and occasional smoking.

## Methods

### Design and participants

Cross-sectional surveys were performed during 2000–2004 among 10^th ^grade pupils in 6 out of 19 counties in Norway, including the capital Oslo, two southern inland counties and three northern counties. Nearly all public and private schools participated. The survey questionnaire was completed during school hours, and standardized explanations on how to complete it were given by trained field personnel. Altogether 15931 pupils (87%) participated. Among pupils completing the questionnaires, 63% lived in cities, with Oslo making up 45% of the study population. The study protocol was approved by the Norwegian Data Inspectorate and by the Regional Committee for Medical Research Ethics.

### Measures

Smoking and use of snuff was measured by questions that separated never, former, occasional and daily users. The question was: "Are you smoking, or have you ever been smoking?" (tick one box only). The response categories were 1) no, never 2) yes, but I have quit 3) yes, occasionally and 4) yes, every day. The question about snuff was worded "Are you using, or have you ever been using snuff, chewing-tobacco or similar products?" with the same response categories as for smoking. In the analysis, both questions on tobacco use were categorized into daily, occasional or no use, with former tobacco users assigned to the no use category. The age for starting smoking was asked (average 13.2 years). No corresponding question was asked for snuff use.

Age was estimated using month and year of birth and date of survey participation. Average age was 15.9 years (range 14.5–18.4 years) and was categorized into quartiles in the analysis.

The parents' marital status was categorized as 1) married/cohabiting 2) unmarried 3) divorced/separated 4) widowed 5) other. The first category was kept as recorded. Remaining categories were combined as "divorced, separated etc" in the analysis.

Parents' country of birth was reported and for the purpose of this study grouped according to Muslim cultural influence. We used three categories: 1) Norwegian parents: at least one parent born in Norway 2) Parents from a Muslim country: both parents born in a country with a Muslim majority population and 3) Parents from other foreign countries: both parents born in other foreign countries or one parent born in a Muslim majority country and one in another foreign country. When information was given for only one of the parents (0.9% of the sample), this information decided to which group the pupil belonged. Muslim cultural background was singled out in the analysis because it is a factor known to affect the use of tobacco, with higher smoking prevalence among men and lower smoking prevalence among women. Muslim religious beliefs have been associated with low smoking prevalence [30,31].

Educational plans were assessed with the question "What is the highest education you are intending to take?" Seven answer categories were collapsed into five: 1) academic studies at higher or medium level: more than (master) or less than (bachelor) four years of college/university 2) upper secondary school, general studies 3) upper secondary school, vocational studies 4) one year at upper secondary school/other plans 5) undecided. In Norway, all pupils are at the same educational level by the age of 15–16 years, as the 10^th ^grade is the last year of compulsory school. After this grade they decide to attend upper secondary school or not. Upper secondary school, general studies, is a pre-requisite for academic studies.

The pupils' consideration of their family economy was assessed by asking if their family, compared to other families in Norway, were probably "very well off," "well off," "in the middle" or "short of money." An urbanization variable was constructed by dividing municipalities into 1) cities (according to administrative definition) or 2) rural areas (non-city municipalities). Partial non-response to questions used in the analyses was generally low (0.5 – 2.3%).

### Statistical analysis

**Figure 1:**
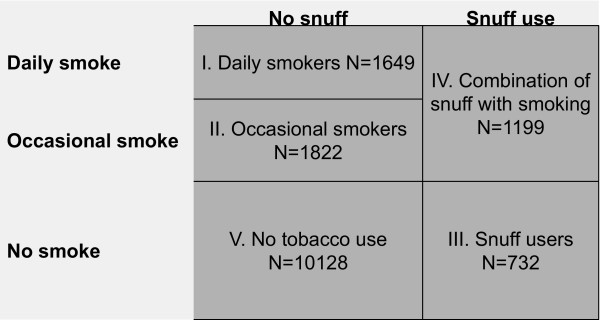
Number of tobacco users and non-users among 15–16 year olds 2000–2004.

We collapsed the six combinations of daily or occasional use of smoke and/or snuff into five groups as shown in figure 1. We did four regression analyses using in turn one of the groups I–IV shown in figure 1 as the outcome variable (coded 1) and regressed it against non-users of tobacco (group V, coded 0), with gender and socio-demographic variables as covariates.

The risk differences for tobacco use were estimated using linear binomial regression. This is a generalized linear model with binomial distribution family and identity link function [32]. In STATA this model can be fitted with the command:

glm y x1 x2 x3, family(binomial) link(identity).

We used the alternative linear regression with a robust variance estimator

regress y x1 x2 x3, robust

The regression coefficient from this model measures the risk difference for tobacco use. As for other linear models, appropriate covariate coding enables the constant term to measure the expected prevalence or risk of tobacco use when all covariates are at their reference categories. The advantage of using risk difference is that differences in absolute risks are shown, in contrast to relative risks or odds ratios. Interaction terms between parents' country of birth and gender were included in all the models.

We also calculated odds ratios (ORs) with 95% confidence intervals (CIs) by using logistic regression and the same models and outcome variables as for the binomial regression.

Data were analysed using STATA, version 9.2 and SPSS, version 14.0.

## Results

Snuff use, daily or occasional, was more common among boys (21.5%) than girls (3.5%) (table 1). This makes snuff use almost as common as smoking for boys. Smoking, daily or occasional, was more common among girls (33.8%) than boys (26.4%). Nearly half of the boys using snuff daily were also smokers, and almost two thirds of occasional snuff users were smokers. About two thirds of boys and girls did not use tobacco in any form.

**Table 1:** Prevalence of tobacco use among 15–16 year olds.

**Boys**				
	**Daily snuff (%)**	**Occas. snuff (%)**	**No snuff (%)**	**Smoke, all (%)**

**Daily smoke (%)**	1.0	6.4	6.4	13.8
**Occasional smoke (%)**	1.8	3.5	7.3	12.6
**No smoke (%)**	3.0	5.8	64.9	73.7

**Snuff use, all (%)**	5.8	15.7	78.6	100.1

**Girls**				

	**Daily snuff (%)**	**Occas. snuff (%)**	**No snuff (%)**	**Smoke, all (%)**

**Daily smoke (%)**	0.0	1.9	14.8	16.7
**Occasional smoke (%)**	0.1	0.8	16.2	17.1
**No smoke (%)**	0.0	0.7	65.5	66.2

**Snuff use, all (%)**	0.1	3.4	96.5	100.0

Per cent 2000–2004

The percentage of daily smokers increased with age for boys, but not for girls (table 2). Boys and girls with single parents had higher smoking prevalence. Daily smoking was strongly associated with educational plans, with the lowest smoking prevalence in the university/college group and among those not yet decided. For both boys and girls, prevalence of daily smoking was highest among those who rated their family economy the lowest.

**Table 2:** Smoking among 15–16 year olds in socio-demographic groups.

	**Boys**					**Girls**				
	N	Daily (%)	Occasionally (%)	No smoke (%)	P*	N	Daily (%)	Occasionally (%)	No smoke (%)	P*

**All participants**	7762	13.8	12.6	73.6		7768	16.8	17.0	66.2	
**Age, years**										

14.5–15.6	1888	11.8	12.5	75.7	<0.037	1991	16.3	18.4	65.2	<0.579
15.6–15.9	1901	13.4	12.2	74.5		1974	17.2	16.5	66.3	
15.9–16.1	1956	14.3	13.1	72.6		1915	16.4	16.3	67.3	
16.1–18.4	1988	15.5	12.7	71.8		1872	17.2	16.8	66.1	

**Parents' marital status**										

Married/cohabiting	5135	10.7	12.4	76.9	<0.000	5152	12.2	16.4	71.4	<0.000
Divorced, separated, etc	2555	19.8	13.1	67.2		2587	25.7	18.4	55.9	

**Parents' country of birth**										

Norway	6737	13.9	12.7	73.5	<0.210	6786	17.9	17.8	64.3	<0.000
Country with majority of Muslims	583	13.6	10.1	76.3		550	6.4	8.4	85.3	
Other foreign countries	330	10.9	13.6	75.5		380	11.8	14.7	73.4	

**Educational plans**										

Academic studies	3320	8.3	12.7	79.0	<0.000	3942	11.5	18.0	70.8	<0.000
Upper secondary school, general studies	436	10.8	12.6	76.6		390	19.7	17.5	62.3	
Upper secondary school, vocat. studies	2420	21.7	13.0	65.3		1700	29.5	15.5	54.9	
One year of upp sec school/other plans	408	20.3	13.2	66.4		303	22.1	21.8	56.1	
Undecided	1053	10.3	11.2	78.5		1355	13.7	15.9	70.5	

**Family economy**										

Very well off	879	13.1	12.7	74.2	<0.000	603	18.6	18.1	63.4	<0.000
Well off	4186	12.2	12.3	75.5		4042	14.1	16.6	69.3	
In between	2347	15.9	12.9	71.2		2736	19.0	17.3	63.7	
Short of money	232	22.0	16.4	61.6		281	28.8	19.9	51.3	

**Urban – rural areas**										

Urban areas	4870	13.0	12.6	74.5	<0.035	4911	16.4	17.5	66.2	<0.244
Rural areas	2892	15.0	12.7	72.3		2857	17.4	16.2	66.3	

Per cent 2000–2004

* p-value for difference between categories within each socio-demographic variable

Snuff use did not vary with age (table 3). Boys and girls with single parents had a higher prevalence of snuff use. Snuff use was rare among adolescents with parents from countries with majority of Muslims. Snuff was negatively associated with educational plans in the same way as smoking and more common in rural than in urban areas.

**Table 3:** Snuff use among 15–16 year olds in socio-demographic groups.

	**Boys**					**Girls**				
	N	Daily (%)	Occasionally (%)	No snuff (%)	P*	N	Daily (%)	Occasionally (%)	No snuff (%)	P*

**All participants**	7762	5.8	15.6	78.6		7768	0.1	3.4	96.5	
**Age, years**										

14.5–15.6	1888	5.6	15.5	78.9	<0.849	1991	0.3	3.3	96.4	<0.058
15.6–15.9	1901	6.4	15.3	78.3		1974	0.1	3.5	96.4	
15.9–16.1	1956	5.3	15.8	79.0		1915	0.0	3.6	96.4	
16.1–18.4	1988	5.6	15.9	78.5		1872	0.0	3.2	96.9	

**Parents' marital status**										

Married/cohabiting	5135	5.0	14.2	80.8	<0.000	5152	0.1	2.9	97.0	<0.004
Divorced, separated, etc	2555	6.9	18.4	74.7		2587	0.1	4.3	95.6	

**Parents' country of birth**										

Norway	6737	6.3	16.7	77.0	<0.000	6786	0.1	3.7	96.2	<0.000
Country with majority of Muslims	583	0.3	6.5	93.1		550	0.0	0.9	99.1	
Other foreign countries	330	1.5	9.4	89.1		380	0.3	1.1	98.7	

**Educational plans**										

Academic studies	3320	4.2	12.5	83.3	<0.000	3942	0.1	2.6	97.3	<0.001
Upper secondary school, general studies	436	5.7	14.2	80.1		390	0.0	3.9	96.2	
Upper secondary school, vocat. studies	2420	7.9	19.6	72.6		1700	0.2	5.1	94.7	
One year of upp. sec. school/other plans	408	7.8	20.3	71.8		303	0.3	4.0	95.7	
Undecided	1053	4.2	15.3	80.5		1355	0.0	3.5	96.5	

**Family economy**										

Very well off	879	7.4	15.5	77.1	<0.164	603	0.2	3.5	96.4	<0.278
Well off	4186	5.7	15.6	78.7		4042	0.1	3.2	96.7	
In between	2347	5.3	15.7	79.0		2736	0.1	3.6	96.4	
Short of money	232	3.0	18.1	78.9		281	0.0	6.1	94.0	

**Urban – rural areas**										

Urban areas	4870	4.9	14.5	80.6	<0.000	4911	0.1	2.4	97.5	<0.000
Rural areas	2892	7.2	17.5	75.3		2857	0.1	5.1	94.8	

Per cent 2000–2004

* p-value for differences between categories within each socio-demographic variable

**Figure 2:**
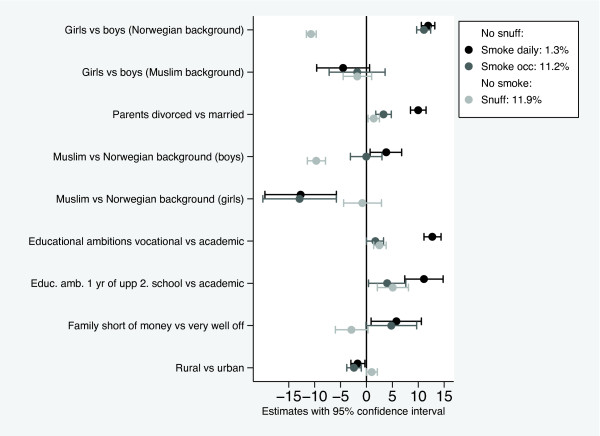
Risk factors expressed as risk difference × 100. The constant term (shown in box) equals expected tobacco use when all covariates are zero*. * Expected tobacco use for a boy in the youngest age quartile, with parents from Norway and living together in an urban area, with academic educational plans and considering his family economy to be very good.

The results from binominal regression models of daily smoking, occasional smoking, and snuff use (daily or occasional) are shown in figure 2 and table 4. The interaction term of gender with parents' country of birth being a country with majority of Muslims was statistically significant, and this interaction term was included in all the models.

**Table 4:** Risk differences calculated from linear binominal regression models with outcome variables I–IV*

	**I. Smoke daily** **no snuff** N = 11351	**II. Smoke occasionally** **no snuff** N = 11539	**III. Snuff** **(daily or occasionally)** ** no smoke** N = 10473	**IV. Smoke and snuff.** **Combination users ** **(daily or occasionally)** N = 10932
**Constant**	1.3	11.2	11.9	13.1
**Gender**				

Boys	0.0	0.0	0.0	0.0
Girls (parents born in Norway)	11.9 (10.6, 13.2)	11.1 (9.7, 12.4)	-10.7 (-11.6, -9.7)	-11.2 (-12.4, -10.1)
Girls (parents born in country w. major. of Muslims)	-4.5 (-9.6, 0.6)	-1.8 (-7.2, 3.6)	-1.8 (-4.5, 1.0)	-5.0 (-8.9, -1.1)

**Age, years**				

Under 15.6	0.0	0.0	0.0	0.0
15.6–15.9	1.1 (-0.6, 2.8)	-0.8 (-2.6, 1.0)	0.4 (-0.9, 1.7)	0.1 (-1.5, 1.7)
15.9–16.1	1.5 (-0.2, 3.2)	-0.5 (-2.3, 1.4)	0.4 (-1.0, 1.7)	0.4 (-1.2, 1.9)
16.1–18.4	2.5 (0.8, 4.3)	-0.3 (-2.1, 1.6)	0.4 (-0.9, 1.7)	0.9 (-0.7, 2.5)

**Parents' marital status**				

Married/cohabiting	0.0	0.0	0.0	0.0
Divorced, separated, etc.	10.0 (8.5, 11.5)	3.3 (1.8, 4.8)	1.4 (0.3, 2.5)	4.5 (3.2, 5.9)

**Parents' country of birth**				

Norway	0.0	0.0	0.0	0.0
Country w. major. of Muslims (boys)	3.8 (0.7, 6.8)	-0.01 (-3.1, 3.0)	-9.7 (-11.4, -7.9)	-9.1 (-11.7, -6.5)
Country w. major. of Muslims (girls)	-12.7 (-19.6, -5.8)	-12.9 (-20.0, -5.8)	-0.8 (-4.4, 2.9)	-2.9 (-8.3, 2.5)
Other foreign countries	-2.4 (-5.2, 0.4)	-2.2 (-5.2, 0.8)	-4.5 (-5.8, -3.1)	-4.0 (-6.1, -1.9)

**Education ambitions**				

Academic studies	0.0	0.0	0.0	0.0
Upper secondary school, general studies	5.3 (2.5, 8.1)	2.6 (-0.4, 0.6)	2.0 (-0.2, 4.2)	2.5 (-0.01, 5.0)
Upper secondary school, vocat. studies	12.7 (11.1, 14.4)	1.7 (0.01, 3.3)	2.5 (1.4, 3.8)	9.1 (7.5, 10.7)
One year of upper sec. school/other plans	11.1 (7.4, 14.8)	4.0 (0.4, 7.6)	5.1 (2.1, 8.1)	6.9 (3.5, 10.3)
Undecided	1.0 (-0.7, 2.6)	-2.3 (-4.1, -0.5)	0.8 (-0.4, 2.0)	1.1 (0.4, 2.5)

**Family economy**				

Very well off	0.0	0.0	0.0	0.0
Well off	-2.3 (-4.4, -0.2)	-2.2 (-4.5, 0.04)	- 1.2 (-3.0, 0.6)	-2.2 (-4.2, -0.1)
In between	-0.5 (-2.8, 1.8)	-0.4 (-2.8, 2.1)	-2.7 (-4.6, -0.9)	-1.9 (-4.1, 0.3)
Short of money	5.8 (0.9, 10.6)	4.8 (-0.1, 9.7)	-2.9 (-6.0, 0.3)	1.4 (-2.9, 5.7)

**Urban-rural**				

Urban	0.0	0.0	0.0	0.0
Rural	-1.7 (-3.0, -0.3)	-2.4 (-3.8, -1.0)	1.0 (-0.04, 2.1)	1.7 (0.5, 2.9)

* The first line shows the constant term which equals expected tobacco use when all covariates are zero. The other lines show risk differences × 100 (with 95% confidence interval) for tobacco use. Zero values are the reference categories

The first line in table 4 shows the constant terms from the model, which is the expected prevalence of tobacco use when all covariates are at their reference values. In other words, a boy in the youngest age quartile, with parents from Norway and living together in an urban area, with academic educational plans and considering his family economy to be very good. The other lines show the risk differences, which are to be added to the constant term when the covariates are not at their reference values. The constant and all model coefficients are multiplied by 100 to increase readability. To calculate the expected prevalence of daily smoking for any covariate pattern, simply add the risk differences in table 4.

**Example**: Boys in the upper quartile of age, with parents living together and born in a country with majority of Muslims, vocational study plans, the family considered short of money, and living in a rural area, have an expected prevalence of daily smoking of 1.3 (constant) + 2.5 (effect of age) + 0 (effect of parents marital status) + 3.8 (effect of Muslim influence for boys) + 12.7 (effect of voc. study plans) + 5.8 (effect of economy) – 1.7 (effect of rural area) = 24.5%.

### Daily smoking

The expected rate of daily smoking was 1.3% for a reference individual (table 4, column I and figure 2). The effect of gender depended on parents' background. Girls with parents born in Norway were 11.9% more likely to smoke than their male counterparts. Girls with parents from Muslim majority countries were 4.5% less likely to smoke than Norwegian boys in the reference category, although this was not significant. Pupils with single parents had a 10% higher risk of daily smoking compared to pupils with parents living together. Pupils planning vocational education had a 12.7% higher risk for daily smoking than those planning an academic education. The factors "single parents" and "vocational education" discriminated clearly between daily smoking and other tobacco use (figure 2). Pupils who considered their families short of money had a 5.8% higher risk of daily smoking than those who considered their families to be very well off. Daily smoking was positively associated with age (+2.5% from 1^st ^to 4^th ^quartile). Pupils living in rural areas had a small, but significantly decreased risk of daily smoking compared to those living in urban areas (-1.7%).

### Occasional smoking

Patterns of occasional smoking were similar to daily smoking, but the associations with education were weaker (table 4, column II and figure 2). Pupils who were undecided about their educational plans had a slightly reduced risk of being an occasional smoker compared to academic oriented pupils. No age differences were found. Differences between urban and rural areas showed similar results as for daily smoking.

### Snuff use

The risk pattern for snuff use was different from smoking. Girls were less likely overall than boys to use snuff, particularly when the parents were born in Norway (table 4, column III and figure 2). Boys with parents from countries with Muslim majority had a 9.7% lower risk of using snuff compared to boys with Norwegian parents. Boys and girls with parents born in other foreign countries also had a lower risk.

Regarding educational plans, the pattern for snuff use was similar to that of occasional smoking (figure 2). Snuff use was weakly associated with single parenthood and family economy. A lower risk of snuff use was found among pupils reporting "in between" family economy, and there was a tendency towards lower risk among the less well off compared with the very well off. No differences were found for age or urbanization.

We intended to include two different models on snuff use, one with daily use and one with occasional use, but the low number of girls using snuff daily limited the use of two separate models. Only small differences in user profiles between occasional and daily snuff users were found for boys. Poor family economy was associated with reduced risk (-4.6%) and single parenthood with increased risk (+2%) of daily, but not occasional use of snuff.

### Combination use of smoking and snuff, versus non-use of tobacco

As for snuff alone, the factors "female" and "parents not born in Norway", whether from a country with Muslim influence or not, were associated with reduced risk of combining smoke and snuff (table 4, column IV). Similarly to smoking, combination use was associated with having divorced parents and plans for vocational study or one year of upper secondary school. The risk for combination use was lower for reported family economy "well off" than for "very well off," and was higher in rural than in urban areas. No age differences were found.

### Relative effects

Alternatively, relative effects can be calculated by using logistic regression. The following ORs (95% CI) may be compared to the risk differences in table 4: For daily smoking, pupils with single parents had an OR of 2.26 (CI 2.01–2.53) compared to those with parents living together. Pupils with ambitions for vocational studies had an OR of 2.89 (CI 2.53–3.29) compared to those with ambitions for academic studies. The OR was 1.37 (CI 0.99–1.89) for family economy "short of money" versus "very well off". The corresponding ORs for occasional smoking were 1.28 (single parents, CI 1.15–1.43), 1.14 (vocational studies, CI 1.00–1.31) and 1.33 (short of money, CI 0.98–1.82). For snuff use the ORs were 1.24 (single parents versus living together, CI 1.05–1.48), 1.48 (vocational versus academic studies, CI 1.22–1.80) and 0.63 ("short of money" versus "very well off," CI 0.35–1.13).

## Discussion

Smoking was more prevalent among adolescents with vocational rather than academic ambitions, single parents, and poor self-reported family economy. Having parents from Muslim counties conferred an increased risk for boys and a decreased risk for girls for daily smoking, compared to adolescents with Norwegian parents. Snuff use and occasional smoking had weaker associations with educational ambitions, family economy and single parenthood than daily smoking. Combination use was associated with single parenthood and vocational study plans. Gender differences are generally found in Scandinavian countries, with higher prevalence of smoking among the girls and higher prevalence of snuff use among the boys [13,27,33].

The strengths of this study are the large and representative study population (nearly 16000 adolescents), high response rate (87%), and a standardized data collection with trained field personnel in all counties.

The main weakness of our study is that all information is self-reported and collected at one point in time [34]. Some pupils may over report their ambitions to attend academic studies and underreport their smoking habits for social desirability reasons, leading to stronger associations in the direction found in our study. Answers, however, were confidential and anonymous, which has been shown to lead to valid self-reported information on adolescent smoking [35-37]. Ethnicity divided only into three groups is a crude measure and was chosen because Muslim cultural influence is a factor known to affect the use of tobacco [30,31]. In the light of the low smoking rates for Muslim women, girls with parents from these countries may underreport their smoking habits due to social desirability [30].

The amount of tobacco used was not asked, which may lead to misclassification. A study from New Zealand showed that 30% of the adolescents reporting to be occasional smokers turned out to be daily smokers when they were asked about the frequency of smoking [37].

We did not have access to parental socioeconomic data in our study. Instead the pupils were asked to give a subjective assessment of the family economy. It is of increasing acceptance to use adolescents' own reports of social status instead of their often inaccurate reports of the SES of their parents [38]. One weakness with the binomial regression model used is that some covariate combinations may give negative smoking prevalence. These combinations are rare or non-existing in the data.

A positive relationship with age was found for smoking, but not for use of snuff or combination use. Worldwide, 19% of 13–15 year old non-smokers reported in 2000–2007 that they might start smoking during the next year [39]. Our analyses showed a higher prevalence of smoking and lower prevalence of combination use in urban than in rural areas. Little is known about the relationship between adolescent smoking and urbanization. Previous studies show the pattern among adults to differ between countries [40-43].

Our study supports previous findings that Muslim identification is associated with high smoking prevalence among men and low prevalence among women [30,31]. Adolescents with different cultural backgrounds have been found to influence each other's health behaviour. For example, in the Oslo part of our study, students with a Norwegian background drank alcohol less frequently when attending schools with a larger proportion of students with a Muslim background [44]. This cross-cultural effect on prevalence of smoking and snuff use seems, however, relatively small compared with overall differences in prevalence of smoking and snuff use between groups of adolescents with different country backgrounds. Further investigation into the cross-cultural effects of tobacco and snuff use is warranted.

Our study showed a negative association between smoking and adolescents' own judgement of family economy, in line with other studies finding a higher risk of tobacco use among adolescents in non-affluent families [45,46]. Our study is also in accordance with other studies showing a higher risk of tobacco use for adolescents with single parents compared with adolescents living with both parents [47-50]. One in four children in Norway are living with only one parent [51], which often implies low income. As the mean age of the pupils in our study was 15.9 years and the initiation age for daily smoking 13.2 years, the probability is relatively high that establishment of the family economy and parents' divorce came before smoking initiation. This may give grounds for a cautious interpretation of these SES-variables as predictors.

Could smoking affect educational ambitions, as well as the opposite being the case? Academic ambitions may be influenced by tobacco use via mediating variables such as attachment to peers with higher or lower academic ambitions. Interestingly, a follow-up study of 16 and 18 year old pupils in Finland found smoking to predict attained educational level. Adolescents' health related lifestyle, rather than health status, with smoking as the strongest predictor, had impact on later educational level. Smoking was considered to be a marker of a broader lifestyle, combined with a rejection of an achievement ideology and the adoption of an anti-school orientation. The number of cigarettes smoked was found to be negatively associated with later educational level [52]. As occasional smokers consume fewer cigarettes than daily smokers, this finding is in line with our finding that occasional smokers had higher educational ambitions than daily smokers, but not as high as non-smokers.

Adolescents' educational ambition has been used as a social indicator by others and is found to correlate with school marks and parents' education level [53,54]. Our results support earlier findings that academic orientation as well as school performance is shown to be closely associated with adolescents' health and health-related behaviour, including smoking [27,55,56]. These associations may be due to parental influence or other factors in the social environment. Peer, teacher and environmental influence may also differ between vocational and academic school-classes [57].

The negative association found between SES and daily smoking was expected. Several other studies confirm these findings among adolescents [19] and it is consistent with Norway being in the late stage of the tobacco epidemic, where the prevalence of smoking continues to decline and gradually reaches a stable minimum level. The decline in prevalence of smoking among lower SES groups lags behind the decline in higher SES groups [20,58].

We expected a positive association between SES and occasional smoking. We found, however, a negative association, although weaker than for daily smoking. A study among 16–18 year old students from Norway found occasional smokers to be in higher academic courses than daily smokers, in line with the differences in educational ambitions in our study [59].

In studies on adults, occasional smokers had higher education levels than daily smokers [24,26]. Our study of a younger age group may indicate a shift to lower SES for occasional smokers, as the tobacco epidemic in general is on the decline. In a Norwegian study from 2006, adolescents rated the "smoker prototype" as less attractive than the "non-smoker-prototype," even amongst regular smokers [60]. Being a non-smoker was associated with being independent, smart and self-confident, indicating that the attitudes towards any type of smoking are slowly changing to be more negative. The spread of attitudes about tobacco use from higher to lower SES levels has been described [18,20,58]. Young people today may be some of the first to adopt a wave of negative attitudes towards occasional smoking, with young people in higher socio-economic groups leading on with tobacco-free practice, and others adopting the negative attitude while still using tobacco.

We had expected less SES difference for snuff use than for daily smoking. This expectation was met regarding educational ambitions and parents' marital status. In a Swedish city, 18 year old students in vocational courses were nearly twice as likely to use snuff as students in academic programmes [27]. Adolescents' own educational orientation was used as a measure, with the results corresponding to our findings using educational ambitions as a measure. Subjective family economy in our study was positively associated with daily snuff use among boys. Our results indicate that snuff use is associated with a higher SES than daily smoking, although snuff use may undergo a similar shift as smoking, starting with decreasing prevalence of use in higher socio-economic groups, and young people being the first to change their habits.

## Conclusion

In a time of rapid changes in tobacco use, in particular among adolescents, it is important to recognize subgroups at high risk. Our study has clearly indicated high-risk for tobacco use among those with ambitions for a vocational rather than academic career, and from less affluent or single parent families. The social and family background differences were largest for daily smoking and less pronounced for occasional smoking and snuff use. There may be an ongoing shift towards lower SES among all groups of tobacco users, including occasional smokers and snuff users. The trends for smoke and smokeless tobacco should be followed, as well as factors contributing to the start and cessation of tobacco use.

## Competing interests

The authors declare that they have no competing interests.

## Authors' contributions

LG analysed the data, drafted the manuscript and contributed to the literature review. HS gave methodological advises and took part in writing the methods section. RH and SGI took the initiative to conduct the study and, contributed to result interpretation and commented on the drafts. RH contributed to the literature review and SGI contributed to the study design, and supervised the drafting of the manuscript. All authors read and approved the final manuscript.

## Pre-publication history

The pre-publication history for this paper can be accessed here:


